# The impact of perceived caregiver anxiety and stress during childhood on late-life depression: evidence from the China Health and Retirement Longitudinal Study

**DOI:** 10.3389/fpsyt.2025.1507566

**Published:** 2025-01-31

**Authors:** Liuyin Jin, Dongdong Wang, Dengxian Yang, Qiong Jin, Mengye Cao, Yuanyuan Li, Jiajie Yang, Guoming Xie, Wenwu Zhang

**Affiliations:** ^1^ Ningbo University Affiliated Kangning Hospital Child and Adolescent Psychiatry Center, Ningbo, Zhejiang, China; ^2^ Science and Education Department, Lishui Second People's Hospital, Lishui, China; ^3^ Neurology Department, Peking University Zibo Hospital, Zibo, Shandong, China; ^4^ Child and Adolescent Psychiatry Center, Ningbo University Affiliated Kangning Hospital, Ningbo, Zhejiang, China; ^5^ Department of Neurology, The Affiliated Lihuili Hospital of Ningbo University, Ningbo University, Ningbo, China

**Keywords:** childhood adversity, perceived caregiver anxiety, perceived caregiver stress, late-life depression, intergenerational mental health, CHARLS

## Abstract

**Introduction:**

This study investigates the long-term impact of perceived caregiver anxiety and stress during childhood on late-life depression. Adverse childhood experiences related to caregiver mental health may significantly influence emotional well-being, and this study utilizes data from the China Health and Retirement Longitudinal Study (CHARLS) to explore these associations.

**Methods:**

CHARLS data were analyzed for individuals who reported perceived caregiver anxiety and stress. Depression was measured using the CES-10 depression scale. Multivariate logistic regression models examined the relationship between caregiver anxiety and stress frequency and late-life depression, adjusting for confounders like socioeconomic status, health behaviors, and demographics.

**Results:**

Childhood exposure to caregiver anxiety and stress significantly increased the risk of depression in later life (p < 0.05), with stronger effects observed among individuals with female caregivers. The risk escalated with the frequency of caregiver anxiety episodes. After adjusting for covariates, the association for male caregivers weakened, highlighting the potential role of other mediators.

**Discussion:**

The results highlight the critical importance of parental mental health, especially maternal anxiety, in mitigating intergenerational mental health risks. Targeted interventions for caregiver mental health, particularly for female caregivers, are crucial. Longitudinal studies are needed to better establish causality and further investigate these mechanisms.

## 1 Introduction

Depression is the third leading cause of disability globally, emerging as a major public health concern that affects individuals across all age groups and exerts profound negative impacts on both physical and mental health. In the United States, the lifetime risk of experiencing a major depressive episode approaches 30% (1, 2). Within this context, experiences within a depressive family environment play a crucial role in shaping the vulnerability and resilience of subsequent generations to depression and anxiety disorders (3). Consequently, research has increasingly focused on the intricate interactions between family dynamics and depression, particularly emphasizing the influence of caregivers’ emotional health on children’s psychological development.

Caregiver anxiety and stress are identified as pivotal factors that may trigger or exacerbate depressive symptoms (4), Moreover, parental depression and anxiety have the potential to be transmitted intergenerationally (5). For instance, Carly J. Johnco and colleagues discovered evidence of the intergenerational transmission of anxiety and depression, noting that parental rejection and low warmth significantly elevate the risk of depression in children (6). Caregivers’ mental health issues, such as anxiety and stress, can adversely affect the family environment, thereby potentially increasing the offspring’s risk of developing depression. Several mechanisms elucidate how caregiver stress impacts children: 1. Neurodevelopmental Impact of the Family Environment: Nicole R. Bush and colleagues found that factors such as family socioeconomic status, family structure and environment, parenting behaviors and interaction styles, parental mental health and functioning, and parental substance use can influence children’s brain development, subsequently affecting their risk of mental illnesses (7); 2. Emotional Contagion: Children may internalize their caregivers’ emotional states, leading to the development of depressive symptoms. Behavioral Modeling: Children may adopt maladaptive coping strategies modeled by their caregivers. For example, Emily L. Robertson and colleagues observed that since the onset of the COVID-19 pandemic, increases in caregivers’ anxiety, anger, sadness/depression, changes in eating and sleeping patterns, diminished hope for the future, and heightened conflict could predict the severity of temper issues, conflicts, and attention-deficit/hyperactivity disorder (ADHD) symptoms in their children one month later (8). 3. Impaired Parenting Practices: Caregiver anxiety can undermine parenting behaviors, resulting in overprotection, inconsistent discipline, or neglect, which in turn can lead to emotional dysregulation and depressive symptoms in children. The deterioration of the parent-child relationship due to caregivers’ emotional unavailability further heightens the likelihood of depression in children (9). 4. Socioeconomic and Environmental Stressors: Caregiver stress is often accompanied by social and economic pressures, which can exacerbate the aforementioned effects by limiting children’s access to resources and opportunities (10, 11). Despite the substantial body of research, most studies have been conducted within Western contexts, primarily focusing on the impact of parental emotions on children’s emotional well-being. There is a notable paucity of research examining the effects of caregiver stress and anxiety experienced during childhood on depression among individuals aged 45 and above. Therefore, investigating the influence of caregiver stress and anxiety during childhood on depression in later life within the Chinese context is of paramount importance.

The China Health and Retirement Longitudinal Study (CHARLS) offers a unique opportunity to explore this relationship within a large, nationally representative sample of Chinese families (12). Using data from CHARLS, this study aims to investigate the association between perceived caregiver anxiety and stress and depressive symptoms in their offspring. Understanding the relationship between experiences of perceived caregiver stress and anxiety during childhood and depression after the age of 45 in the Chinese context is crucial for developing targeted interventions. Such interventions can address familial mental health issues and mitigate the intergenerational transmission of mental disorders. Consequently, this study leverages caregiver mental health assessments from the CHARLS database, in conjunction with the Center for Epidemiologic Studies Depression Scale (CESD-10) (13), to examine the long-term impact of perceived caregiver anxiety and stress on adult depression. By analyzing how caregivers’ mental health influences their children’s emotional well-being, this research contributes to the expanding knowledge base. It also provides insights for strategic public health initiatives aimed at alleviating the burden of depression through family-centered approaches.

## 2 Methods

### 2.1 Study population

The China Health and Retirement Longitudinal Study (CHARLS) is administered by the National Institute of Development and Research in China and was initiated in 2011. Data collection occurs biennially, with a total of five waves completed by 2020. The inaugural baseline survey was conducted from June 2011 to March 2012, encompassing 17,705 participants. Utilizing a Probability Proportional to Size (PPS) sampling method, participants were randomly selected from 28 provinces, 150 counties/districts, and 450 villages/residential committees. Data collection was performed through Computer-Assisted Personal Interviewing (CAPI) conducted in participants’ homes. The study received ethical approval from the Biomedical Ethics Review Committee of Peking University (IRB00001052–11015), and all participants provided informed consent.

**Figure 1:**
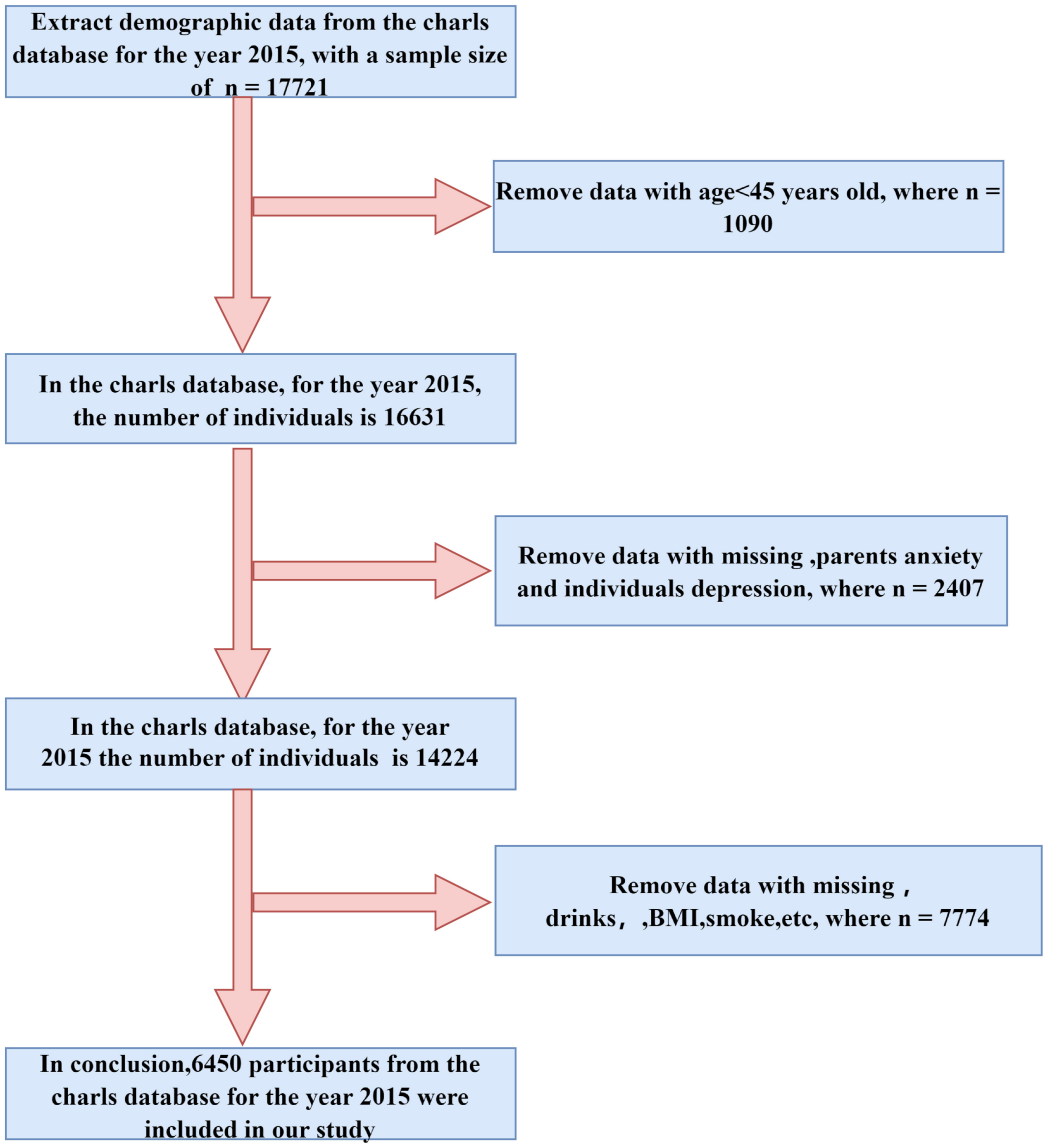
The flowchart of inclusion and exclusion for the 2015 follow-up data.

CHARLS employs a longitudinal design with stratified sampling and PPS methods to ensure a nationally representative sample of Chinese families. It targets residents aged 45 and above, thereby offering comprehensive insights into the demographic and health status of China’s urban and rural elderly populations. To investigate the association between perceived caregiver stress and anxiety and the incidence of depression in offspring, the sample was restricted to individuals with available family baseline information in 2014, matched with corresponding data from 2015 based on participant IDs. Participants with missing family information in 2014, absent depression data in 2015, or incomplete covariate information in 2015 were excluded. Ultimately, 6,450 participants were included in the subsequent analyses. The specific criteria for inclusion and exclusion are detailed in **Figure 1**.

### 2.2 Depression assessment

Depressive symptoms were evaluated using the Center for Epidemiologic Studies Depression Scale (CES-D-10) within the CHARLS database. The CES-D-10 is a validated instrument for measuring depressive symptoms among elderly populations in China. It comprises 10 items, each with four response options: “Rarely or none of the time” (<1 day), “Some or a little of the time” (1-2 days), “Occasionally or a moderate amount of time” (3-4 days),” Most or all of the time” (5-7 days). Each response is scored from 0 to 3, resulting in a total score range of 0 to 30, with higher scores indicating greater severity of depressive symptoms. A cutoff score of ≥10 is employed to identify individuals exhibiting significant depressive symptoms.

### 2.3 Assessment of caregiver stress and anxiety

In 2014, CHARLS incorporated a life course questionnaire that included a section on caregiver mental health. Two specific items were selected to assess caregiver anxiety and stress:A. “When you were a child, did your female caregiver often feel tense or anxious?”A little of the time (0); Some of the time (1); Good part of the time (2); Most of the time (3). B. “When you were a child, did your male caregiver often feel tense or anxious?” A little of the time (0); Some of the time (1); Good part of the time (2); Most of the time (3). Responses of 2, 3, or 4 were indicative of the individual having experienced caregiver stress and anxiety during childhood. These responses were scored accordingly, with 0 points assigned to responses indicating minimal or no stress/anxiety, and higher scores reflecting greater levels of caregiver stress and anxiety. The total scores for male and female caregivers were calculated separately and subsequently combined to form an overall caregiver anxiety score. Additionally, analyses were conducted to separately evaluate the impact of female and male caregiver anxiety and stress on the prevalence of depression in offspring during adulthood.

### 2.4 Covariate assessment

This study primarily investigates the relationship between perceived caregiver anxiety/stress and offspring depression, recognizing that depression is influenced by a multitude of factors. To account for potential confounders, the following covariates were included in the analysis:Demographic Variables: Gender, age, residence (urban/rural), education level, marital status, and race. Health-Related Factors: Body Mass Index (BMI), obesity, hypertension, diabetes.Lifestyle Factors: Smoking and alcohol consumption.Socioeconomic Indicators: Family economic status during childhood.These covariates were selected based on their established associations with the incidence of depression, ensuring a comprehensive adjustment for potential confounding variables in the analysis of the primary relationship under investigation (14, 15).

### 2.5 Statistical analysis

Continuous variables are presented as means ± standard deviations (SD) or medians with interquartile ranges (IQR), while categorical variables are expressed as percentages. To rigorously examine the association between individual depression and perceived caregiver stress and anxiety, we employed multivariable logistic regression analyses. Three distinct logistic models were utilized to calculate the odds ratios (OR) and 95% confidence intervals (CI) for the relationship between caregiver stress and anxiety and depression in offspring: Model 1: Crude (unadjusted) association. Model 2: Adjusted for age, sex, education, location, marital status, BMI, obesity, and nationality.Model 3: Further adjusted for smoking, alcohol consumption, hypertension, diabetes mellitus (DM), and childhood financial status, in addition to the covariates included in Model 2.Subgroup analyses were conducted to explore the relationship between caregiver stress and anxiety and offspring depression across various demographic and health-related strata. All statistical analyses were performed using R version 4.3.2.

## 3 Results

### 3.1 Baseline characteristics of depressed individuals

**Table 1** delineates the baseline characteristics of participants who met the inclusion criteria during the 2015 follow-up survey. The non-depressed group had an average age of 60.87 ± 9.19 years, whereas the depressed group averaged 61.22 ± 8.95 years (p = 0.13), indicating no significant difference in age distribution between the two cohorts. In terms of Body Mass Index (BMI), the depressed group exhibited a slightly lower mean BMI of 23.98 ± 4.13 compared to the non-depressed group’s mean BMI of 24.24 ± 3.70, a difference that reached statistical significance (p = 0.01). Caregiver anxiety scores were substantially higher in the depressed group (1.29 ± 1.84) than in the non-depressed group (0.87 ± 1.56), with the difference being highly statistically significant (p < 0.0001). Gender distribution revealed that females constituted a significantly larger proportion of the depressed group (73.91%) compared to the non-depressed group (61.99%, p < 0.0001). Similarly, the depressed group had a higher percentage of unmarried individuals (16.71%) compared to the non-depressed group (11.18%, p < 0.0001). Educational attainment analysis showed that a greater proportion of the depressed group had primary or lower education levels (78.18%) compared to the non-depressed group (64.03%, p < 0.0001). Rural residency was more prevalent in the depressed group (70.06%) than in the non-depressed group (59.35%, p < 0.0001). Regarding smoking status, the depressed group had a lower proportion of current smokers (17.80%) compared to the non-depressed group (20.85%, p < 0.0001). Additionally, underweight status was more common in the depressed group (6.74%) compared to the non-depressed group (4.48%, p < 0.001).Caregiver characteristics indicated significant differences between the depressed and non-depressed groups for both male and female caregivers. Specifically, higher caregiver anxiety and stress scores were associated with the depressed group. Furthermore, childhood financial status analysis revealed that a higher percentage of the depressed group reported poorer economic conditions, with 28.14% considering themselves “far worse off than others” compared to 19.18% in the non-depressed group (p < 0.0001). No significant differences were observed between the two groups concerning ethnicity, alcohol consumption, hypertension, or diabetes mellitus. Detailed data are presented in **Table 1**.

**Table 1:** Basic information table.

variable	total (n=6450)	normal (n=4062)	depression (n=2388)	statistic	p.value
**age**	61.00 ± 9.10	60.87 ± 9.19	61.22 ± 8.95	1.51	0.13
**BMI**	24.14 ± 3.87	24.24 ± 3.70	23.98 ± 4.13	-2.47	0.01
**anxious_score**	1.03 ± 1.68	0.87 ± 1.56	1.29 ± 1.84	9.18	<0.0001
**sex**				95.28	<0.0001
female	4283 (66.40)	2518 (61.99)	1765 (73.91)		
male	2167 (33.60)	1544 (38.01)	623 (26.09)		
**marital_status**				39.62	<0.0001
married	5597 (86.78)	3608 (88.82)	1989 (83.29)		
non-married	853 (13.22)	454 (11.18)	399 (16.71)		
**education**				149.46	<0.0001
college and higher	90 (1.40)	79 (1.94)	11 (0.46)		
Elementary school and below	4468 (69.27)	2601 (64.03)	1867 (78.18)		
High school	1892 (29.33)	1382 (34.02)	510 (21.36)		
**location**				73.72	<0.0001
rural	4084 (63.32)	2411 (59.35)	1673 (70.06)		
urban	2366 (36.68)	1651 (40.65)	715 (29.94)		
**nationality**				0.15	0.70
Ethnic Minority	482 (7.47)	308 (7.58)	174 (7.29)		
Non-Ethnic Minority	5968 (92.53)	3754 (92.42)	2214 (92.71)		
**smoke**				24.02	<0.0001
Current smoker	1272 (19.72)	847 (20.85)	425 (17.80)		
Former smoker	674 (10.45)	464 (11.42)	210 (8.79)		
Never	4504 (69.83)	2751 (67.73)	1753 (73.41)		
**drink**				0.18	0.68
no	4912 (76.16)	3086 (75.97)	1826 (76.47)		
yes	1538 (23.84)	976 (24.03)	562 (23.53)		
**hypertension**				0.36	0.55
no	4675 (72.48)	2955 (72.75)	1720 (72.03)		
yes	1775 (27.52)	1107 (27.25)	668 (27.97)		
**DM**				1.41	0.23
no	5406 (83.81)	3422 (84.24)	1984 (83.08)		
yes	1044 (16.19)	640 (15.76)	404 (16.92)		
**obesity**				19.07	<0.001
low weight	343 (5.32)	182 (4.48)	161 (6.74)		
normal	2966 (45.98)	1849 (45.52)	1117 (46.78)		
obesity	941 (14.59)	602 (14.82)	339 (14.20)		
over weight	2200 (34.11)	1429 (35.18)	771 (32.29)		
**female_guardian_factor**				92.48	<0.0001
A little of the time	4388 (68.03)	2932 (72.18)	1456 (60.97)		
Some of the time	1012 (15.69)	579 (14.25)	433 (18.13)		
Good part of the time	579 (8.98)	310 (7.63)	269 (11.26)		
Most of the time	471 (7.30)	241 (5.93)	230 (9.63)		
**male_guardian_factor**				73.53	<0.0001
A little of the time	4687 (72.67)	3092 (76.12)	1595 (66.79)		
Some of the time	860 (13.33)	500 (12.31)	360 (15.08)		
Good part of the time	525 (8.14)	279 (6.87)	246 (10.30)		
Most of the time	378 (5.86)	191 (4.70)	187 (7.83)		
**financial_situation_factor**				96.95	<0.0001
A lot better off than them	61 (0.95)	46 (1.13)	15 (0.63)		
Somewhat better off than them	543 (8.42)	373 (9.18)	170 (7.12)		
Same as them	3371 (52.26)	2261 (55.66)	1110 (46.48)		
Somewhat worse off than them	1024 (15.88)	603 (14.84)	421 (17.63)		
A lot worse off than them	1451 (22.50)	779 (19.18)	672 (28.14)		

The bold text within the table denotes specific variables that are of particular importance to the study's methodology and findings.

### 3.2 Logistic regression of perceived caregiver stress and anxiety and late-life depression

We found a significant association between perceived caregiver stress and anxiety and late-life depression. Although the association decreased as covariates were added, it remained statistically significant. In the crude risk model, perceived caregiver stress and anxiety scores were significantly positively correlated with child depression, with a 95% CI of 1.61 (1.45, 1.79), p < 0.0001. In Model 1, the 95% CI was 1.59 (1.43, 1.78), p < 0.0001. In Model 2, the 95% CI was 1.49 (1.33, 1.66), p < 0.0001 (detailed data can be found in **Table 2**).

**Table 2:** Logistic regression of anxiety and stress among caregivers and depression in late life.

Anxious_score						P
Crude model		Model 1		Model 2		
Depression	95%CI	P	95%CI	P	95%CI	
anxious_score	1.61 (1.45,1.79)	<0.0001	1.59 (1.43,1.78)	<0.0001	1.49 (1.33,1.66)	<0.0001

We further performed regression analysis on perceived caregiver stress and anxiety and child depression, separating male and female caregivers. Among male caregivers, the association with child depression was significant in the crude and partially adjusted models. However, after full adjustment for covariates, the association was no longer significant. In contrast, for female caregivers, the association remained significant across all three models.

For male caregivers, we used “A little of the time” as the reference group. In the crude model, the 95% CI for “Some of the time” was 1.4 (1.20, 1.62), p < 0.0001, for “Good part of the time” was 1.71 (1.43, 2.05), p < 0.0001, and for “Most of the time” was 1.9 (1.54, 2.34), p < 0.0001, with p for trend < 0.0001. In Model 1, the 95% CI for “Some of the time” was 1.4 (1.20, 1.62), p < 0.0001, for “Good part of the time” was 1.61 (1.33, 1.94), p < 0.0001, and for “Most of the time” was 1.82 (1.47, 2.26), p < 0.0001, with p for trend < 0.0001. In Model 2, the 95% CI for “Some of the time” was 1.01 (0.83, 1.23), p = 0.9, for “Good part of the time” was 1.14 (0.89, 1.47), p = 0.31, and for “Most of the time” was 1.22 (0.90, 1.67), p = 0.2, with p for trend = 0.16 (more information can be found in **Table 3**).

**Table 3:** Logistic regression of anxiety and stress among male caregivers and depression in late life.

Male_guardian_factor						P
Crude model		Model 1		Model 2		
Depression	95%CI	P	95%CI	P	95%CI	
A little of the time	ref		ref		ref	
Some of the time	1.4 (1.20,1.62)	<0.0001	1.4 (1.20,1.63)	<0.0001	1.01 (0.83,1.23)	0.9
Good part of the time	1.71 (1.43,2.05)	<0.0001	1.61 (1.33,1.94)	<0.0001	1.14 (0.89,1.47)	0.31
Most of the time	1.9 (1.54,2.34)	<0.0001	1.82 (1.47,2.26)	<0.0001	1.22 (0.90,1.67)	0.2
p for trend (character2integer)		<0.0001		<0.0001		0.16

For female caregivers, the risk of child depression showed a significant positive correlation with perceived caregiver stress and anxiety, particularly in groups more frequently monitored by female caregivers, where depression risk was higher. Even after controlling for various confounding factors, the association remained significant, although with a relatively lower risk ratio. This suggests that female caregiving may play an important role in depression risk. Similarly to male caregivers, we used “A little of the time” as the reference group. In the crude model, the 95% CI for “Some of the time” was 1.51 (1.31, 1.73), p < 0.0001, for “Good part of the time” was 1.75 (1.47, 2.08), p < 0.0001, and for “Most of the time” was 1.92 (1.59, 2.33), p < 0.0001, with p for trend < 0.0001. In Model 1, the 95% CI for “Some of the time” was 1.56 (1.35, 1.80), p < 0.0001, for “Good part of the time” was 1.68 (1.41, 2.01), p < 0.0001, and for “Most of the time” was 1.86 (1.53, 2.26), p < 0.0001, with p for trend < 0.0001. In Model 2, the 95% CI for “Some of the time” was 1.49 (1.24, 1.79), p = 0.9, for “Good part of the time” was 1.39 (1.09, 1.77), p = 0.01, and for “Most of the time” was 1.47 (1.11, 1.96), p = 0.01, with p for trend < 0.0001 (more information can be found in **Table 4**).

**Table 4:** Logistic regression of female caregivers and depression in late life.

Female_guardian_factor						P
Crude model		Model 1		Model 2		
Depression	95%CI	P	95%CI	P	95%CI	
A little of the time	ref		ref		ref	
Some of the time	1.51 (1.31,1.73)	<0.0001	1.56 (1.35,1.80)	<0.0001	1.49 (1.24,1.79)	<0.0001
Good part of the time	1.75 (1.47,2.08)	<0.0001	1.68 (1.41,2.01)	<0.0001	1.39 (1.09,1.77)	0.01
Most of the time	1.92 (1.59,2.33)	<0.0001	1.86 (1.53,2.26)	<0.0001	1.47 (1.11,1.96)	0.01
p for trend (character2integer)		<0.0001		<0.0001		<0.001

Length.

### 3.3 Subgroup analysis of perceived caregiver stress and anxiety and the association with late-life depression

**Figure 2:**
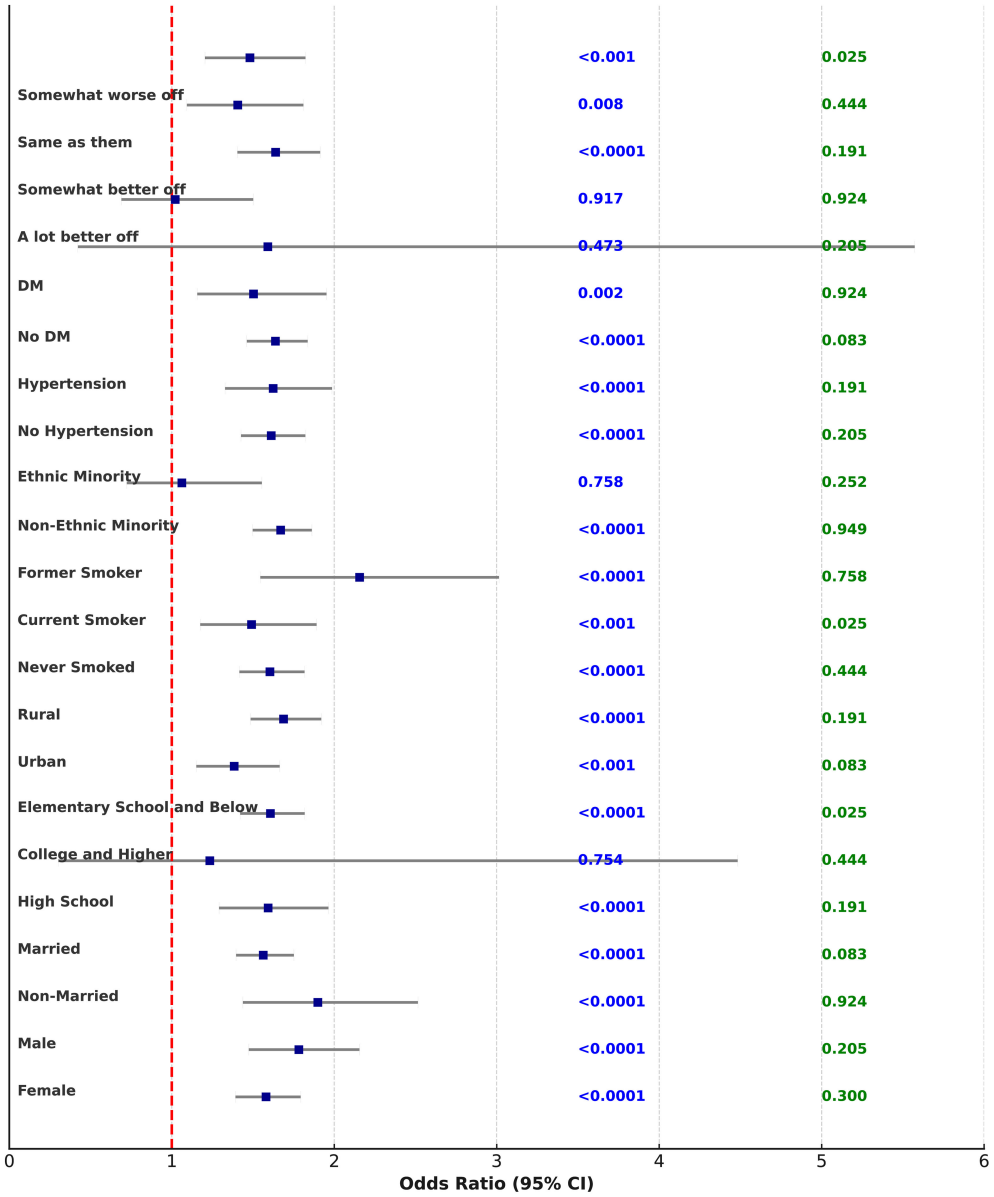
The forest plot of subgroup analysis on guardian stress and anxiety in relation to children’s depression.

We conducted a subgroup analysis on the association between perceived caregiver anxiety and child depression to explore potential interactions based on different demographic and health characteristics. The analysis results are as follows: in the gender subgroup, the p for interaction = 0.3, with the hazard ratio (HR) being 1.580 (95% CI: 1.392, 1.793), P < 0.0001 in females, and HR = 1.782 (95% CI: 1.474, 2.155), P < 0.0001 in males. For marital status, p for interaction = 0.205, with HR = 1.899 (95% CI: 1.438, 2.515), P < 0.0001 in unmarried individuals and HR = 1.563 (95% CI: 1.396, 1.751), P < 0.0001 in married individuals. Regarding education level, p for interaction = 0.924, with HR = 1.593 (95% CI: 1.291, 1.964), P < 0.0001 in individuals with high school education, HR = 1.234 (95% CI: 0.301, 4.483), P = 0.754 in those with college education or higher, and HR = 1.607 (95% CI: 1.421, 1.818), P < 0.0001 in those with elementary education or less. For geographical location, p for interaction = 0.083, with HR = 1.384 (95% CI: 1.151, 1.664), P < 0.001 in urban areas and HR = 1.688 (95% CI: 1.485, 1.920), P < 0.0001 in rural areas. Regarding smoking status, p for interaction = 0.191, with HR = 1.604 (95% CI: 1.416, 1.817), P < 0.0001 in never-smokers, HR = 1.491 (95% CI: 1.175, 1.891), P < 0.001 in current smokers, and HR = 2.156 (95% CI: 1.545, 3.014), P < 0.0001 in former smokers. For alcohol consumption, p for interaction = 0.444, with HR = 1.653 (95% CI: 1.466, 1.864), P < 0.0001 in non-drinkers and HR = 1.503 (95% CI: 1.216, 1.858), P < 0.001 in drinkers. In terms of ethnicity, p for interaction = 0.025, with HR = 1.670 (95% CI: 1.498, 1.862), P < 0.0001 in non-minorities and HR = 1.062 (95% CI: 0.723, 1.554), P = 0.758 in minorities. Regarding hypertension, p for interaction = 0.949, with HR = 1.612 (95% CI: 1.426, 1.822), P < 0.0001 in those without hypertension and HR = 1.624 (95% CI: 1.328, 1.986), P < 0.0001 in those with hypertension. For diabetes status, p for interaction = 0.554, with HR = 1.638 (95% CI: 1.462, 1.836), P < 0.0001 in those without diabetes and HR = 1.503 (95% CI: 1.157, 1.952), P = 0.002 in those with diabetes. For childhood economic status, p for interaction = 0.252, with HR = 1.591 (95% CI: 0.422, 5.573), P = 0.473 in those who considered their financial status “much better than others,” HR = 1.021 (95% CI: 0.690, 1.501), P = 0.917 in those who considered themselves “slightly better than others,” HR = 1.639 (95% CI: 1.404, 1.913), P < 0.0001 in those who considered themselves “the same as others,” HR = 1.406 (95% CI: 1.093, 1.810), P = 0.008 in those who considered themselves “slightly worse than others,” and HR = 1.481 (95% CI: 1.204, 1.823), P < 0.001 in those who considered themselves “much worse than others.” Detailed data are shown in **Figure 2** and **Supplementary Table 1**.

**Figure 3:**
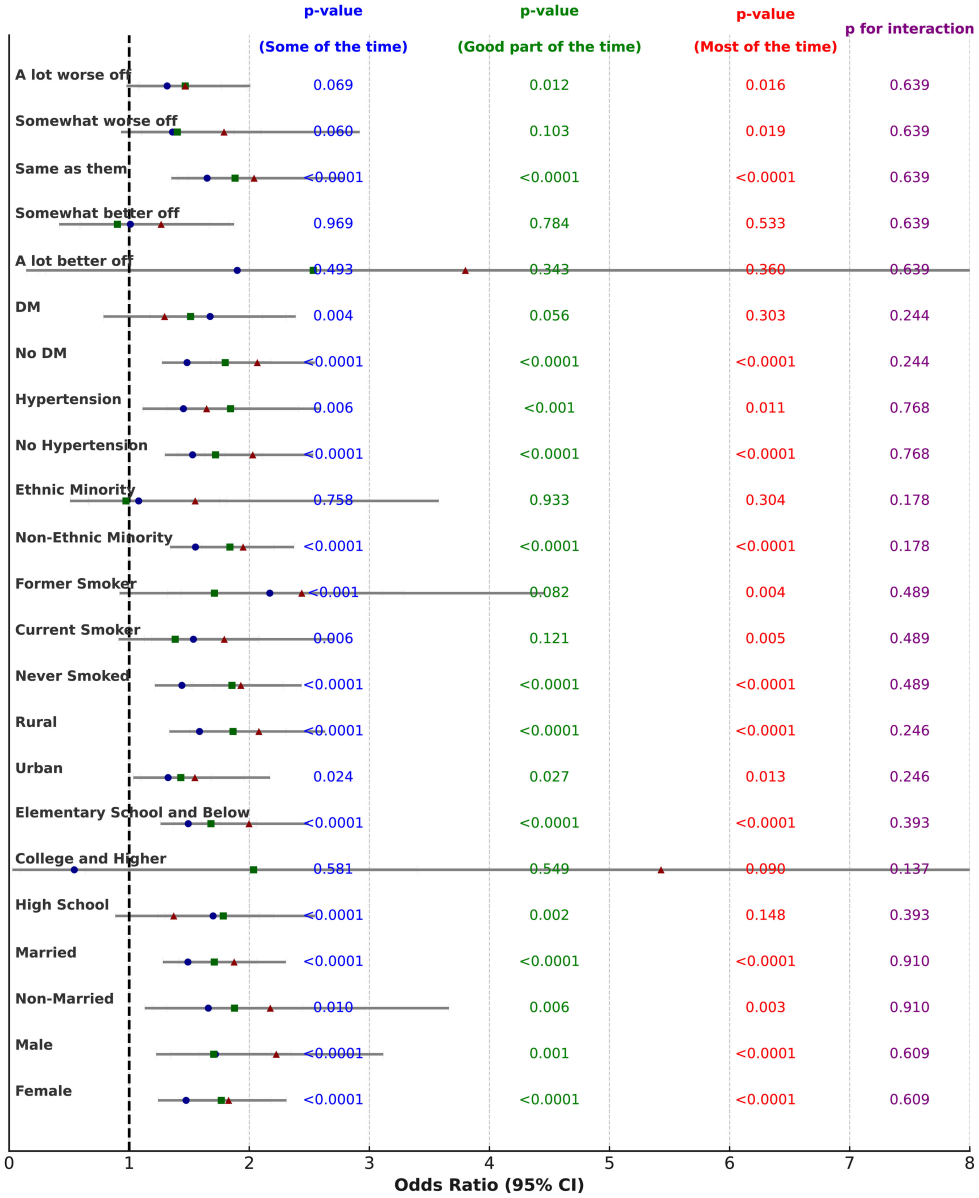
The forest plot of subgroup analysis on male guardian stress and anxiety in relation to children’s depression. This figure is a forest plot of the subgroup analysis on male guardian stress and anxiety in relation to the risk of children’s depression. It shows how different levels of female guardian stress and anxiety (“Some of the time,” “Good part of the time,” and “Most of the time”) affect the risk of depression in children. Each entry displays the Odds Ratio (OR) and its 95% confidence interval (CI) for various subgroups. Different shapes and colors represent the results for the three time periods: blue circles represent “Some of the time,” green squares represent “Good part of the time,” and red triangles represent “Most of the time.” • X-axis: Represents the OR (Odds Ratio), with the dashed line indicating OR = 1. If the 95% confidence interval of the OR does not include 1, it indicates that the factor has a statistically significant effect on the outcome. • p-value: The p-values for each time period are displayed in the blue, green, and red columns, indicating the statistical significance of maternal anxiety on children’s depression for each time period. • p for interaction: The purple column on the far right shows the p-value for interaction, which tests for significant differences between subgroups.

We analyzed the association between perceived caregiver anxiety and depression risk in children for different subgroups, focusing on male and female caregivers, and stratified by gender, marital status, education level, geographical location, smoking, alcohol consumption, ethnicity, hypertension, diabetes, and financial status. Overall, the association between perceived caregiver stress and late-life depression risk was significant in most subgroups, particularly regarding gender, marital status, education level, geographical location, smoking, and alcohol consumption. While not all interactions were statistically significant, the general trend indicated that the longer the father’s anxiety persisted, the higher the risk of depression in the child. The subgroup analysis for male caregivers showed the following results: regarding gender, p for interaction = 0.67. For females, as perceived male caregiver stress and anxiety increased, the risk of depression in children also increased, with HRs of 1.663 (95% CI: 1.333, 2.075), P < 0.0001 for the “Good part of the time” group and 2.035 (95% CI: 1.566, 2.650), P < 0.0001 for the “Most of the time” group. For males, the HRs were 1.879 (95% CI: 1.352, 2.595), P < 0.001 and 1.799 (95% CI: 1.240, 2.589), P = 0.002 for these groups, respectively. For marital status, p for interaction = 0.596, with HR = 2.690 (95% CI: 1.534, 4.868), P < 0.001 in the “Most of the time” group for unmarried individuals and HR = 1.777 (95% CI: 1.413, 2.234), P < 0.0001 for married individuals. Regarding education level, p for interaction = 0.324, with HR = 1.701 (95% CI: 1.069, 2.663), P = 0.022 for high school graduates and HR = 1.839 (95% CI: 1.443, 2.348), P < 0.0001 for those with elementary education or less. For geographical location, p for interaction = 0.538, with HR = 1.610 (95% CI: 1.086, 2.365), P = 0.016 in urban residents and HR = 1.958 (95% CI: 1.520, 2.527), P < 0.0001 in rural residents. For smoking status, p for interaction = 0.654, with HR = 2.079 (95% CI: 1.608, 2.691), P < 0.0001 in never-smokers and HR = 2.025 (95% CI: 0.991, 4.057), P = 0.048 in former smokers. For alcohol consumption, p for interaction = 0.993, with HR = 1.871 (95% CI: 1.476, 2.373), P < 0.0001 in non-drinkers and HR = 1.994 (95% CI: 1.262, 3.152), P = 0.003 in drinkers. Regarding ethnicity, p for interaction = 0.324, with HR = 1.938 (95% CI: 1.560, 2.408), P < 0.0001 in non-minorities and HR = 1.357 (95% CI: 0.540, 3.299), P = 0.502 in minorities. For hypertension, p for interaction = 0.922, with HR = 1.931 (95% CI: 1.515, 2.460), P < 0.0001 in those without hypertension and HR = 1.818 (95% CI: 1.185, 2.786), P = 0.006 in those with hypertension. For diabetes, p for interaction = 0.228, with HR = 2.027 (95% CI: 1.612, 2.548), P < 0.0001 in those without diabetes. Regarding childhood financial status, p for interaction = 0.466, with HR = 1.966 (95% CI: 1.378, 2.797), P < 0.001 in those who considered their financial status the same as others and HR = 1.329 (95% CI: 0.948, 1.864), P = 0.099 in those who considered themselves much worse than others. More data can be found in **Figure 3** and **Supplementary Table 2**.

**Figure 4:**
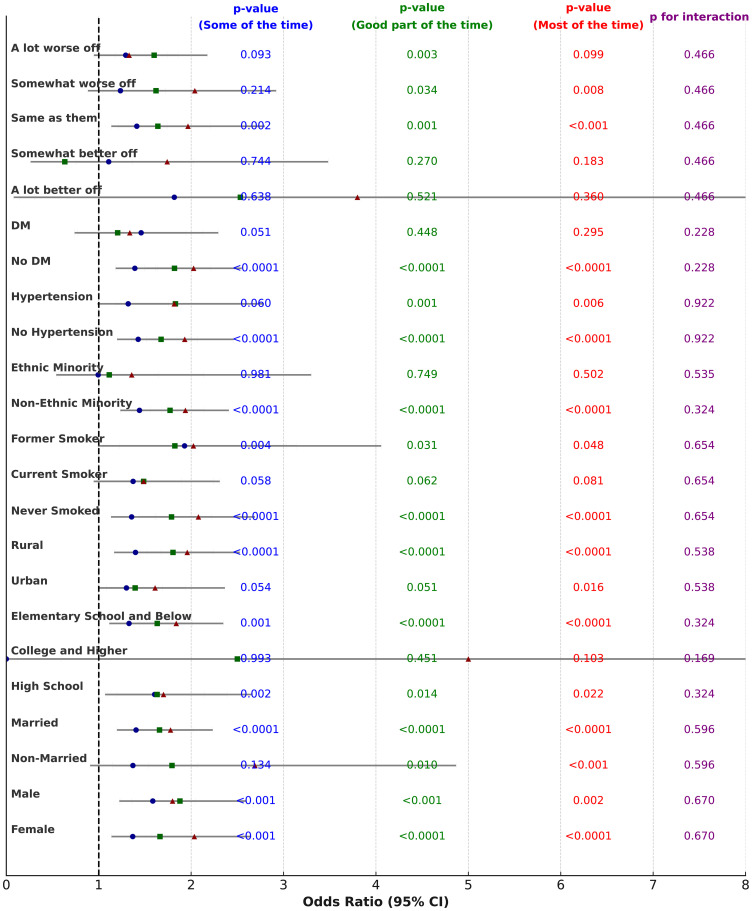
The forest plot of subgroup analysis female on guardian stress and anxiety in relation to children’s depression. This figure is a forest plot of the subgroup analysis on female guardian stress and anxiety in relation to the risk of children’s depression. It shows how different levels of female guardian stress and anxiety (“Some of the time,” “Good part of the time,” and “Most of the time”) affect the risk of depression in children. Each entry displays the Odds Ratio (OR) and its 95% confidence interval (CI) for various subgroups. Different shapes and colors represent the results for the three time periods: blue circles represent “Some of the time,” green squares represent “Good part of the time,” and red triangles represent “Most of the time.” • X-axis: Represents the OR (Odds Ratio), with the dashed line indicating OR = 1. If the 95% confidence interval of the OR does not include 1, it indicates that the factor has a statistically significant effect on the outcome. • p-value: The p-values for each time period are displayed in the blue, green, and red columns, indicating the statistical significance of maternal anxiety on children’s depression for each time period. • p for interaction: The purple column on the far right shows the p-value for interaction, which tests for significant differences between subgroups.

The subgroup analysis of perceived female caregiver stress and anxiety and the association with late-life depression showed similar results to the analysis for male caregivers. Regarding gender, p for interaction = 0.609, with the depression risk in late-life increasing significantly as female caregiver stress and anxiety increased. In the “Good part of the time” and “Most of the time” groups, HRs were 1.767 (95% CI: 1.431, 2.181), P < 0.0001 and 1.828 (95% CI: 1.447, 2.310), P < 0.0001 for females. For males, the highest depression risk was observed in the “Most of the time” group, with HR = 2.225 (95% CI: 1.581, 3.116), P < 0.0001. For marital status, p for interaction = 0.91, with HR = 2.175 (95% CI: 1.312, 3.663), P = 0.003 in the “Most of the time” group for unmarried individuals and HR = 1.874 (95% CI: 1.522, 2.305), P < 0.0001 for married individuals. For education level, p for interaction = 0.393, with HR = 1.371 (95% CI: 0.884, 2.083), P = 0.148 for high school graduates and HR = 1.998 (95% CI: 1.602, 2.495), P < 0.0001 for those with elementary education or less. Regarding geographical location, p for interaction = 0.246, with HR = 1.547 (95% CI: 1.093, 2.173), P = 0.013 in urban residents and HR = 2.080 (95% CI: 1.646, 2.632), P < 0.0001 in rural residents. For smoking status, p for interaction = 0.489, with HR = 1.930 (95% CI: 1.530, 2.436), P < 0.0001 in never-smokers and HR = 2.437 (95% CI: 1.321, 4.464), P = 0.004 in former smokers. Regarding alcohol consumption, p for interaction = 0.661, with HR = 1.848 (95% CI: 1.487, 2.295), P < 0.0001 in non-drinkers and HR = 2.205 (95% CI: 1.469, 3.317), P < 0.001 in drinkers. For ethnicity, p for interaction = 0.178, with HR = 1.949 (95% CI: 1.601, 2.373), P < 0.0001 in non-minorities and HR = 1.550 (95% CI: 0.660, 3.578), P = 0.304 in minorities. For hypertension, p for interaction = 0.768, with HR = 2.029 (95% CI: 1.626, 2.532), P < 0.0001 in those without hypertension and HR = 1.645 (95% CI: 1.120, 2.409), P = 0.011 in those with hypertension. For diabetes, p for interaction = 0.244, with HR = 2.067 (95% CI: 1.679, 2.545), P < 0.0001 in those without diabetes. Regarding childhood financial status, p for interaction = 0.639, with HR = 2.040 (95% CI: 1.485, 2.797), P < 0.0001 in those who considered their financial status the same as others and HR = 1.467 (95% CI: 1.073, 2.008), P = 0.016 in those who considered themselves much worse than others. More data can be found in **Figure 4** and **Supplementary Table 3**.

## 4 Discussion

Numerous studies have demonstrated that adverse childhood experiences significantly heighten the risk of developing mental health disorders in adulthood. These findings have been consistently confirmed in both developed and developing countries (14, 16). The CHARLS database has been utilized in several papers to explore the impact of adverse childhood experiences on depression in later life (15, 17, 18). However, research focusing on the specific effects of individual childhood adversities on future depression remains scarce. To date, only a handful of studies have examined the influence of parental mental disorders during childhood on depression in later life, as well as the role of childhood socioeconomic status in predicting mid-to-late-life depression (19, 20). Given these gaps, our study aims to investigate the impact of perceived caregiver anxiety and stress experienced during childhood on depression in later life, with the goal of deepening the understanding of how specific childhood adversities influence mental health in adulthood. Our findings reveal that individuals perceived caregiver anxiety and stress during childhood are more susceptible to depression in middle and late adulthood. Notably, the stress and anxiety of female caregivers continued to exert a significant influence on depression even after adjusting for potential confounding variables. Additionally, we observed a clear dose-response relationship: as the frequency of perceived caregiver stress and anxiety increased, so did the individual’s risk of depression. Interestingly, in the case of female caregivers, the risk of depression decreased when the frequency of stress and anxiety was classified as “Good part of the time” in Model 2. Through extensive subgroup analyses, we further identified that caregiver anxiety is strongly associated with an increased risk of depression in offspring, particularly across variables such as gender, marital status, residence, and smoking or drinking behaviors.

A family history of mood disorders and anxiety is a well-established risk factor for developing mood disorders. Research shows that children of parents with mental illnesses often exhibit alterations in brain regions related to cognitive and emotional processing. Jennifer V. A. Kemp and colleagues examined brain structural changes in children of parents with major depressive disorder (MDD), anxiety, or bipolar disorder (BD). They found that parental BD was significantly associated with reduced caudate volume, inferior frontal gyrus thickness, and anterior cingulate cortex thickness in offspring. In contrast, parental MDD was linked to alterations in the offspring’s amygdala and hippocampus volumes, fusiform gyrus thickness, and temporoparietal thickness. Moreover, early life adversity (ELA) often results in structural changes to the brain, particularly in areas like the hippocampus and amygdala, and dysregulates the stress response system, notably the hypothalamic-pituitary-adrenal (HPA) axis. Children who endure ELA are at a significantly higher risk of developing mental health disorders, such as depression, in adulthood. This aligns with our findings, which suggest that childhood adversity, including parental anxiety and stress, may increase the likelihood of future depression (21–23). Although the link between parental mental health and child brain structure has been extensively studied, research specifically examining the effects of parental anxiety on brain structure is limited. One study found that parental anxiety symptoms are associated with structural changes in the offspring’s amygdala, while another study observed a similar relationship with the hippocampus (24). Additional research has highlighted the influence of maternal prenatal anxiety on children’s brain development. Elevated maternal anxiety levels during mid-pregnancy have been associated with reduced gray matter density in children aged 6-9, increasing their vulnerability to neurodevelopmental disorders, psychiatric conditions, and cognitive impairments (25, 26). Other studies have demonstrated a connection between maternal anxiety and amygdala development in children. Specifically, maternal anxiety during mid-pregnancy was negatively correlated with functional connectivity between the left amygdala and bilateral parietal clusters. Furthermore, higher maternal anxiety levels were associated with stronger negative connectivity. Postnatal maternal anxiety was positively correlated with increased amygdala volume in children (22, 27). Consistent with these findings, our study shows that perceived caregiver anxiety and stress, particularly that of female caregivers, have a significant impact on depression in late life. The higher the frequency of caregiver anxiety and stress, the more pronounced the effect, reinforcing the current body of evidence.Interestingly, research on the effects of perceived male caregivers’ stress and anxiety on late-life depression remains limited. Some studies have investigated the impact of parental depression, anxiety, and stress on children’s health, such as in cases of type 1 diabetes, revealing that parental stress and anxiety increase the likelihood of depression in children. Moreover, research has suggested that the relationship between fathers’ negative emotions and adolescents’ self-efficacy is mediated by the fathers’ perceptions of their children’s self-efficacy (28, 29). Alice Wickersham and colleagues conducted a meta-analysis of 14 studies examining the association between paternal psychopathology and adolescent depression and anxiety, concluding that paternal depression is associated with both adolescent depression and anxiety, while findings regarding other paternal mental health disorders are less conclusive (30). Alana M. Rogers and colleagues, in their observation of 1,539 mothers and 793 partners, found that maternal perinatal depressive and anxiety symptoms may have adverse effects on infant development, while paternal depressive and anxiety symptoms were not significantly associated with such outcomes (31)Our study similarly found that, after adjusting for several confounding factors, the effect of perceived male caregivers’ stress and anxiety on late-life depression was no longer statistically significant, in line with previous findings.

In conclusion, our study provides further evidence that childhood perceived to caregiver anxiety and stress, particularly from female caregivers, can significantly increase the risk of depression in later life. The findings underscore the importance of considering the nuanced impacts of caregiver mental health on long-term psychological outcomes and highlight the need for targeted interventions to mitigate these risks from an early age.This study focuses on the anxiety and stress experienced by caregivers during childhood, exploring the long-term impact of perceived caregiver anxiety on depression in later life. The findings indicate that this effect is enduring, with the anxiety and stress of female caregivers having a particularly strong influence on individuals. Therefore, it is crucial to prioritize the mental health of both parents, especially female caregivers, in the context of family caregiving. Early detection of parental anxiety, coupled with targeted psychological support, may be an important strategy to mitigate the transmission of mental health issues across generations. Despite the strengths of the study, such as a large nationally representative sample and comprehensive adjustment for confounders, several limitations should be acknowledged. First, the cross-sectional nature of the data makes it impossible to establish a causal relationship between parental anxiety and offspring depression. Longitudinal studies are needed in the future to better understand the temporal relationship between parental and offspring mental health, as well as the potential bidirectional influences. Second, while we adjusted for many confounding factors, unmeasured variables, such as genetic predispositions or specific parenting styles, may still influence the observed associations. Additionally, the data were collected via retrospective self-report questionnaires, which may be subject to recall bias. Future research should further investigate the differential mechanisms by which male and female caregivers’ anxiety affects offspring depression. Examining father-child interactions, gender differences in caregiving responsibilities, and the cultural factors that shape parenting behaviors could provide deeper insights into effective interventions for these relationships. Despite the large sample size, the study did not use validated scales or conduct direct interviews with caregivers to assess anxiety and stress symptoms. Additionally, individuals with depression may have a negative perception of themselves, their experiences (including childhood memories), and others, which could introduce recall bias in the reported caregiver anxiety and stress. The retrospective self-report nature of the data may introduce recall bias, and the cross-sectional analysis limits causal inferences. Future longitudinal studies are needed to better understand the temporal relationships and mechanisms underlying the observed associations.

## 5 Conclusion

This study demonstrates that perceived caregiver anxiety and stress during childhood significantly increases the risk of depression in later life, with a particularly pronounced influence from female caregivers. Furthermore, as the frequency of perceived caregiver anxiety and stress increases, so does the risk of depression. This research highlights the crucial role of parental mental health in the psychological development of children, particularly the long-term effects of female caregivers’ anxiety on their offspring. Although the impact of perceived male caregivers’ anxiety and stress on offspring depression diminishes after adjusting for covariates, future studies should further explore the differential effects of male and female caregivers, as well as the underlying mechanisms influencing their impact on offspring depression.

## Data availability statement

Publicly available datasets were analyzed in this study. This data can be found at: https://charls.ccer.edu.cn/charls/.
