# Supplementary material for: The impact of perceived caregiver anxiety and stress during childhood on late-life depression: evidence from the China Health and Retirement Longitudinal Study
Source: Front Psychiatry. 2025 Jan 31;16:1507566. doi: 10.3389/fpsyt.2025.1507566 (PMC11825812; doi:10.3389/fpsyt.2025.1507566)
Supplement: Supplementary file 1 [file Table1.docx]

Stable1 The subgroup analysis results of guardian stress and anxiety in relation to children's depression.

| **character** | **95% CI** | **p** | **p for interaction** |
| --- | --- | --- | --- |
| **sex** |  |  | 0.3 |
| female | 1.580(1.392,1.793) | <0.0001 |  |
| male | 1.782(1.474,2.155) | <0.0001 |  |
| **marital_status** |  |  | 0.205 |
| non-married | 1.899(1.438,2.515) | <0.0001 |  |
| married | 1.563(1.396,1.751) | <0.0001 |  |
| **education** |  |  | 0.924 |
| High school | 1.593(1.291,1.964) | <0.0001 |  |
| college and higher | 1.234(0.301,4.483) | 0.754 |  |
| Elementary school and below | 1.607(1.421,1.818) | <0.0001 |  |
| **location** |  |  | 0.083 |
| urban | 1.384(1.151,1.664) | <0.001 |  |
| rural | 1.688(1.485,1.920) | <0.0001 |  |
| **smoke** |  |  | 0.191 |
| Never | 1.604(1.416,1.817) | <0.0001 |  |
| Current smoker | 1.491(1.175,1.891) | <0.001 |  |
| Former smoker | 2.156(1.545,3.014) | <0.0001 |  |
| **drink** |  |  | 0.444 |
| no | 1.653(1.466,1.864) | <0.0001 |  |
| yes | 1.503(1.216,1.858) | <0.001 |  |
| **nationality** |  |  | 0.025 |
| Non-Ethnic Minority | 1.670(1.498,1.862) | <0.0001 |  |
| Ethnic Minority | 1.062(0.723,1.554) | 0.758 |  |
| **hypertension** |  |  | 0.949 |
| no | 1.612(1.426,1.822) | <0.0001 |  |
| yes | 1.624(1.328,1.986) | <0.0001 |  |
| **DM** |  |  | 0.554 |
| yes | 1.503(1.157,1.952) | 0.002 |  |
| no | 1.638(1.462,1.836) | <0.0001 |  |
| **financial_situation_factor** |  |  | 0.252 |
| A lot better off than them | 1.591(0.422,5.573) | 0.473 |  |
| Somewhat better off than them | 1.021(0.690,1.501) | 0.917 |  |
| Same as them | 1.639(1.404,1.913) | <0.0001 |  |
| Somewhat worse off than them | 1.406(1.093,1.810) | 0.008 |  |
| A lot worse off than them | 1.481(1.204,1.823) | <0.001 |  |

Stable2 The subgroup analysis results of male guardian stress and anxiety in relation to children's depression.

| **character** | **A little of the time** | **Some of the time** | **p** | **Good part of the time** | **p** | **Most of the time** | **p** | **p for trend(character2integer)** | **p for interaction** |
| --- | --- | --- | --- | --- | --- | --- | --- | --- | --- |
| **sex** |  |  |  |  |  |  |  |  | 0.67 |
| female | ref | 1.368(1.138,1.643) | <0.001 | 1.663(1.333,2.075) | <0.0001 | 2.035(1.566,2.650) | <0.0001 | <0.0001 |  |
| **male** | ref | 1.585(1.223,2.045) | <0.001 | 1.879(1.352,2.595) | <0.001 | 1.799(1.240,2.589) | 0.002 | <0.0001 |  |
| **marital_status** |  |  |  |  |  |  |  |  | 0.596 |
| non-married | ref | 1.370(0.907,2.072) | 0.134 | 1.793(1.151,2.814) | 0.010 | 2.690(1.534,4.868) | <0.001 | <0.0001 |  |
| married | ref | 1.404(1.197,1.646) | <0.0001 | 1.658(1.357,2.025) | <0.0001 | 1.777(1.413,2.234) | <0.0001 | <0.0001 |  |
| **education** |  |  |  |  |  |  |  |  | 0.324 |
| High school | ref | 1.605(1.194,2.146) | 0.002 | 1.632(1.098,2.394) | 0.014 | 1.701(1.069,2.663) | 0.022 | <0.001 |  |
| college and higher | ref | 0.000(NA) | 0.993 | 2.500(0.116, 22.444) | 0.451 | 5.000(0.592, 35.007) | 0.103 | 0.169 |  |
| Elementary school and below | ref | 1.328(1.115,1.582) | 0.001 | 1.633(1.325,2.014) | <0.0001 | 1.839(1.443,2.348) | <0.0001 | <0.0001 |  |
| **location** |  |  |  |  |  |  |  |  | 0.538 |
| urban | ref | 1.300(0.992,1.693) | 0.054 | 1.394(0.993,1.941) | 0.051 | 1.610(1.086,2.365) | 0.016 | 0.001 |  |
| rural | ref | 1.398(1.167,1.674) | <0.001 | 1.804(1.447,2.248) | <0.0001 | 1.958(1.520,2.527) | <0.0001 | <0.0001 |  |
| **smoke** |  |  |  |  |  |  |  |  | 0.654 |
| Never | ref | 1.356(1.133,1.622) | <0.001 | 1.788(1.436,2.226) | <0.0001 | 2.079(1.608,2.691) | <0.0001 | <0.0001 |  |
| Current smoker | ref | 1.372(0.987,1.897) | 0.058 | 1.486(0.975,2.244) | 0.062 | 1.486(0.946,2.310) | 0.081 | 0.009 |  |
| Former smoker | ref | 1.929(1.221,3.027) | 0.004 | 1.824(1.047,3.135) | 0.031 | 2.025(0.991,4.057) | 0.048 | 0.001 |  |
| **drink** |  |  |  |  |  |  |  |  | 0.993 |
| no | ref | 1.405(1.181,1.668) | <0.001 | 1.721(1.391,2.129) | <0.0001 | 1.871(1.476,2.373) | <0.0001 | <0.0001 |  |
| yes | ref | 1.379(1.028,1.843) | 0.031 | 1.688(1.186,2.396) | 0.003 | 1.994(1.262,3.152) | 0.003 | <0.0001 |  |
| **nationality** |  |  |  |  |  |  |  |  | 0.324 |
| Non-Ethnic Minority | ref | 1.441(1.234,1.683) | <0.0001 | 1.772(1.466,2.141) | <0.0001 | 1.938(1.560,2.408) | <0.0001 | <0.0001 |  |
| Ethnic Minority | ref | 0.994(0.590,1.647) | 0.981 | 1.114(0.565,2.138) | 0.749 | 1.357(0.540,3.299) | 0.502 | 0.535 |  |
| **hypertension** |  |  |  |  |  |  |  |  | 0.922 |
| no | ref | 1.427(1.199,1.695) | <0.0001 | 1.675(1.356,2.068) | <0.0001 | 1.931(1.515,2.460) | <0.0001 | <0.0001 |  |
| yes | ref | 1.319(0.986,1.758) | 0.060 | 1.830(1.275,2.626) | 0.001 | 1.818(1.185,2.786) | 0.006 | <0.0001 |  |
| **DM** |  |  |  |  |  |  |  |  | 0.228 |
| yes | ref | 1.459(0.997,2.130) | 0.051 | 1.205(0.739,1.944) | 0.448 | 1.336(0.770,2.294) | 0.295 | 0.099 |  |
| no | ref | 1.391(1.183,1.633) | <0.0001 | 1.820(1.495,2.215) | <0.0001 | 2.027(1.612,2.548) | <0.0001 | <0.0001 |  |
| **financial_situation_factor** |  |  |  |  |  |  |  |  | 0.466 |
| A lot better off than them | ref | 1.818(0.080,20.767) | 0.638 | 1.818(0.231,10.676) | 0.521 | 20937501.309(0.000,NA) | 0.991 | 0.15 |  |
| Somewhat better off than them | ref | 1.108(0.586,2.027) | 0.744 | 0.633(0.263,1.365) | 0.270 | 1.741(0.754,3.928) | 0.183 | 0.643 |  |
| Same as them | ref | 1.412(1.137,1.748) | 0.002 | 1.640(1.213,2.210) | 0.001 | 1.966(1.378,2.797) | <0.001 | <0.0001 |  |
| Somewhat worse off than them | ref | 1.235(0.883,1.723) | 0.214 | 1.620(1.037,2.532) | 0.034 | 2.040(1.206,3.483) | 0.008 | <0.001 |  |
| A lot worse off than them | ref | 1.292(0.958,1.743) | 0.093 | 1.600(1.179,2.176) | 0.003 | 1.329(0.948,1.864) | 0.099 | 0.004 |  |

Stable3 The subgroup analysis results of female guardian stress and anxiety in relation to children's depression.

| **character** | **A little of the time** | **Some of the time** | **p** | **Good part of the time** | **p** | **Most of the time** | **p** | **p for trend(character2integer)** | **p for interaction** |
| --- | --- | --- | --- | --- | --- | --- | --- | --- | --- |
| **sex** |  |  |  |  |  |  |  |  | 0.609 |
| female | ref | 1.474(1.240,1.751) | <0.0001 | 1.767(1.431,2.181) | <0.0001 | 1.828(1.447,2.310) | <0.0001 | <0.0001 |  |
| male | ref | 1.718(1.347,2.187) | <0.0001 | 1.704(1.225,2.353) | 0.001 | 2.225(1.581,3.116) | <0.0001 | <0.0001 |  |
| **marital_status** |  |  |  |  |  |  |  |  | 0.91 |
| non-married | ref | 1.659(1.130,2.442) | 0.010 | 1.877(1.197,2.964) | 0.006 | 2.175(1.312,3.663) | 0.003 | <0.0001 |  |
| married | ref | 1.489(1.281,1.730) | <0.0001 | 1.709(1.412,2.068) | <0.0001 | 1.874(1.522,2.305) | <0.0001 | <0.0001 |  |
| education |  |  |  |  |  |  |  |  | 0.393 |
| High school | ref | 1.699(1.303,2.206) | <0.0001 | 1.784(1.239,2.546) | 0.002 | 1.371(0.884,2.083) | 0.148 | <0.001 |  |
| college and higher | ref | 0.543(0.028, 3.398) | 0.581 | 2.036(0.097,16.460) | 0.549 | 5.429(0.635,38.812) | 0.090 | 0.137 |  |
| Elementary school and below | ref | 1.492(1.261,1.764) | <0.0001 | 1.681(1.371,2.061) | <0.0001 | 1.998(1.602,2.495) | <0.0001 | <0.0001 |  |
| location |  |  |  |  |  |  |  |  | 0.246 |
| urban | ref | 1.324(1.035,1.687) | 0.024 | 1.430(1.037,1.958) | 0.027 | 1.547(1.093,2.173) | 0.013 | <0.001 |  |
| rural | ref | 1.586(1.335,1.883) | <0.0001 | 1.865(1.507,2.308) | <0.0001 | 2.080(1.646,2.632) | <0.0001 | <0.0001 |  |
| smoke |  |  |  |  |  |  |  |  | 0.489 |
| Never | ref | 1.439(1.214,1.705) | <0.0001 | 1.856(1.510,2.281) | <0.0001 | 1.930(1.530,2.436) | <0.0001 | <0.0001 |  |
| Current smoker | ref | 1.535(1.127,2.084) | 0.006 | 1.383(0.912,2.073) | 0.121 | 1.792(1.183,2.700) | 0.005 | <0.001 |  |
| Former smoker | ref | 2.171(1.428,3.291) | <0.001 | 1.710(0.920,3.107) | 0.082 | 2.437(1.321,4.464) | 0.004 | <0.001 |  |
| drink |  |  |  |  |  |  |  |  | 0.661 |
| no | ref | 1.550(1.317,1.821) | <0.0001 | 1.811(1.479,2.216) | <0.0001 | 1.848(1.487,2.295) | <0.0001 | <0.0001 |  |
| yes | ref | 1.395(1.058,1.836) | 0.018 | 1.574(1.105,2.234) | 0.011 | 2.205(1.469,3.317) | <0.001 | <0.0001 |  |
| nationality |  |  |  |  |  |  |  |  | 0.178 |
| Non-Ethnic Minority | ref | 1.552(1.341,1.795) | <0.0001 | 1.839(1.532,2.207) | <0.0001 | 1.949(1.601,2.373) | <0.0001 | <0.0001 |  |
| Ethnic Minority | ref | 1.079(0.658,1.750) | 0.758 | 0.973(0.508,1.809) | 0.933 | 1.550(0.660,3.578) | 0.304 | 0.477 |  |
| hypertension |  |  |  |  |  |  |  |  | 0.768 |
| no | ref | 1.528(1.297,1.800) | <0.0001 | 1.719(1.401,2.107) | <0.0001 | 2.029(1.626,2.532) | <0.0001 | <0.0001 |  |
| yes | ref | 1.452(1.111,1.894) | 0.006 | 1.844(1.309,2.597) | <0.001 | 1.645(1.120,2.409) | 0.011 | <0.0001 |  |
| DM |  |  |  |  |  |  |  |  | 0.244 |
| yes | ref | 1.674(1.173,2.387) | 0.004 | 1.511(0.986,2.307) | 0.056 | 1.295(0.786,2.112) | 0.303 | 0.021 |  |
| no | ref | 1.482(1.273,1.725) | <0.0001 | 1.800(1.484,2.180) | <0.0001 | 2.067(1.679,2.545) | <0.0001 | <0.0001 |  |
| financial_situation_factor |  |  |  |  |  |  |  |  | 0.639 |
| A lot better off than them | ref | 1.900(0.239, 11.299) | 0.493 | 2.533(0.303, 17.408) | 0.343 | 3.800(0.142,101.965) | 0.360 | 0.18 |  |
| Somewhat better off than them | ref | 1.010(0.599,1.669) | 0.969 | 0.902(0.417,1.832) | 0.784 | 1.266(0.587,2.618) | 0.533 | 0.731 |  |
| Same as them | ref | 1.649(1.351,2.009) | <0.0001 | 1.882(1.407,2.512) | <0.0001 | 2.040(1.485,2.797) | <0.0001 | <0.0001 |  |
| Somewhat worse off than them | ref | 1.361(0.986,1.877) | 0.060 | 1.401(0.932,2.102) | 0.103 | 1.790(1.101,2.920) | 0.019 | 0.004 |  |
| A lot worse off than them | ref | 1.316(0.978,1.770) | 0.069 | 1.467(1.088,1.978) | 0.012 | 1.467(1.073,2.008) | 0.016 | 0.002 |  |
